# High school suicide in South Africa: teachers’ knowledge, views and training needs

**DOI:** 10.1186/s12889-015-1599-3

**Published:** 2015-03-14

**Authors:** Hilda N Shilubane, Arjan ER Bos, Robert AC Ruiter, Bart van den Borne, Priscilla S Reddy

**Affiliations:** Department of Advanced Nursing Science, University of Venda, Private Bag X 5050, Thohoyandou, South Africa; Department of Clinical Psychology, Open University, Heerlen, the Netherlands; Department of Work and Social Psychology, Maastricht University, Maastricht, the Netherlands; Department of Health Promotion, Maastricht University, Maastricht, the Netherlands; Population Health, Health Systems and Innovations (PHHSI) Research Programme, Human Sciences Research Council, Capetown, South Africa

**Keywords:** Suicide, Teachers, Knowledge, Mental health, School, Prevention

## Abstract

**Background:**

Suicidal ideation and attempted suicide are a huge problem in South Africa, especially in the rural areas. Previous research has emphasized the importance of the ability of school professionals to identify young people who are at risk of committing suicide. The objectives of this study were to assess the knowledge of teachers with regard to identifying the warning signs of suicidal behaviour, assessing the type of information they give to students in the class after a suicide of one of their class mates, and assessing their views and training needs on the prevention of suicidal behaviour in students.

**Methods:**

Five focus group discussions were conducted with 50 high school teachers in Limpopo Province, South Africa. All focus group discussions were audio-taped, transcribed verbatim, and then analysed using an inductive approach.

**Results:**

The results demonstrate that teachers lack knowledge of the warning signs of suicidal behaviour among students. They also report that they do not know how to support students in the event of attempted or completed suicide of another student. The school curriculum is perceived as lacking information on suicide and suicidal behaviour.

**Conclusions:**

Teachers in Limpopo Province need to be trained to identify students at risk, and to respond to situations by referring individuals at risk to appropriate mental health professionals. School-based suicide prevention programmes that are based on theory and evidence should be developed. These programmes should include teacher training to help teachers to identify symptoms of psychosocial problems that might lead to suicide, develop their skills in handling such problems, and help students to cope with their emotions after a suicide incident in the class or at school.

## Background

Suicide remains a significant social and public health problem [1,2]. In 1998, suicide constituted 1.8% of the total disease burden globally, and is estimated to rise to 2.4% by 2020 [3]. Suicidal behaviour is complex. The process ranges from suicidal ideation, that can be communicated through verbal or non-verbal means, to the planning of suicide, attempting suicide, and in the worst case, actual suicide [4]. Worldwide, suicide has been found to be one of the three leading causes of death among those in the most productive age group (15-44) and the second leading cause of death in the 15-19 years age group [5,6].

Suicide impacts on the most vulnerable of the world’s population and places a large burden on low- and middle-income countries that are often ill-equipped to meet the general and mental health needs of their populations. Services are scarce in these countries as they have low budgetary allocations for health in general and for mental health in particular. As a result, there are few sustained efforts and activities that focus on suicide prevention on a scale necessary to reduce the number of lives lost to suicide [7].

Studies of personal and environmental factors related to suicide ideation and attempt among young people have been conducted in South Africa. One of these studies, conducted [8] in Cape Town, examined the correlates of suicide risk among secondary school students and found that anger-related control problems, low self-esteem, perceived stress and unmet school goals were predictors of suicidal behaviour. Another study on environmental factors and adolescent suicidal behaviours, conducted in Limpopo Province, found that family conflict was a significant correlate for suicidal behaviours [9]. In another, qualitative study of adolescents in Limpopo, it was concluded that a lack of knowledge of the availability of counsellors, conflict in interpersonal relationships, perceived accusations of negative behaviour, inadequate social support, past family and peer suicide attempts, as well as poor living circumstances were associated with attempted suicide [10]. A subsequent quantitative study among the same population demonstrated that suicide ideation is prevalent among these adolescents. Perceived lack of social support and negative feelings about the family and behavioural factors such as forced sexual intercourse and physical violence of partners were positively correlated with the risk of suicidal ideation [11,12] and [13] also found that adolescents who attempted or completed suicide demonstrated warning signs in advance. They tended to talk about suicide, have sleeping and eating problems, withdraw from friends, give away prized possessions, lose interest in their personal appearance, use alcohol or drugs, and take unnecessary risks. Furthermore some studies have demonstrated that drug use is commonly associated with suicide [14-16].

Addressing problems related to suicide and suicide prevention and treatment should be seen as a multi-professional issue. Those who do self-harm might seek help from, or be referred to, a variety of professionals in the community such as teachers, social workers, community mental health nurses, general practitioners, and psychiatrists. This suggests that there is a need to explore the level of knowledge and understanding that staff members of schools have of adolescent self-harm behaviour. There is also a need to gauge teachers’ ability to identify impending acts of student self-harm, prevent school conditions that might lead to student self-harm, and cope with uncertainties and emotions of students after a suicidal incident in the class or school [17]. In response to the problem of suicide, many schools acknowledge that suicide issues are often unavoidable and school professionals are increasingly accepting the role of “gatekeeper” in dealing with suicidal students. As students disclose information about themselves in their daily interactions through conversations with peers, their writing, and general behaviour towards school staff, they provide a gateway for teachers to detect warning signs of suicidal behaviour and to offer support or refer them for professional help [5,18].

It seems as if suicide prevention programmes might reduce the incidents of student suicide at schools. However, before an effective intervention programme can be developed and implemented in high school settings, the knowledge and skills base of teachers needs to be assessed. Some literature emphasises the importance of the ability of school professionals to identify young people who are developing suicide risk behaviours [19], but unfortunately teachers are often neglected in the discussion of adolescent suicide. Teachers should possess accurate knowledge of suicide and be capable of referring a student to relevant services [20,21].

Research in South Africa has neglected the involvement of teachers in studies on adolescent suicide. Teachers’ knowledge of suicidal behaviour is important in preventing adolescent suicide as well as in providing support to peers and the wider school environment following a suicide attempt. The purpose of the study was to assess the knowledge, skills and training needs of teachers concerning adolescent suicidal behaviour. Although Wyman et al. [22] demonstrated that many vulnerable youth will not confide to adults their distress even if they are suicidal, the suicide rate of students might be reduced if teachers were educated about students’ suicide behaviour and taught skills through well designed evidence- and theory-based intervention programmes, as has been shown by previous suicide prevention strategies for physicians [23]. In addition, teachers’ knowledge of warning signs of suicidal behaviour could assist in the identification and referral of students at risk to available counsellors or psychologists. It should be noted that counselling services are available at the health institutions not Limpopo schools.

Furthermore, during the interviews teachers’ indicated that their failure to recognize warning signs of suicidal behaviour contributed to students’ death by suicide. Should they have known that social withdrawal and talking about suicide through social network were warning signs of suicidal behaviour, they would have referred the students for professional help.

## Methods

### Design and participants

A qualitative research design was used with five focus group discussions, using an in-depth interview technique. Participants included 50 high school teachers from Limpopo Province, South Africa. The five focus groups were limited to ten participants and included both males and females (27 females and 23 males).

An interview guide that aimed to gather information on suicidal behaviour, content taught to students, and actions taken by teachers after the suicide of a student was developed. The selection of questions for the focus group discussions was based on previous studies conducted by the research team and literature consulted. Key themes that emerged were teachers’ knowledge about suicide, teachers’ awareness of available mental health care and resources for prevention, opinions on suicide prevention, ways to prevent suicide, and resources available to deal with students at risk and survivors of attempted suicide. The interview schedule was prepared in both the local language of the participants (Xitsonga) as well as English, so that the preference of each group could be accommodated.

### Sampling and procedure

The principals of the seven schools in which a learner was reported to have died by suicide in the period between January and December 2011 were approached to request permission to conduct the study. These schools were selected as a result of the research team’s knowledge of the deaths as a result of their previous studies on suicide behaviour (reference anonymized to ensure blind review). Each of the principals was personally visited by the first author, in order to provide a detailed explanation of the aims of the study. Upon agreement and permission being granted to conduct the research in the schools, the class teachers of the deceased learners were approached. Reasons for the study were explained and consent was obtained for participation in the focus group interviews. Formal appointments detailing times and venues for the full discussions were made. Of the seven schools approached, one principal refused, citing political sensitivities as the reason while teachers from another school refused to participate because of their exam commitments and work schedules. Five government schools participated in the study.

All school teachers in the five high schools who knew or taught the learner before death by suicide were invited to participate in the focus group discussions. Interviews were conducted from April to June 2012. Focus group discussions were held at a time and place convenient to the individual participants and the host organisations. Participants participated at their own free will.

### The focus group procedure

The first author, a professional nurse, moderated the focus groups. At the beginning of each session, the moderator introduced the project and explained the purpose of the focus group. The discussions were audio-taped, and lasted from 60 to 90 minutes. Participants were informed that participation was voluntary and that they were free to withdraw from the discussion at any time without penalties. All participants who agreed to participate in the study completed the focus group sessions. A total of five focus group discussions proved to be sufficient to reach data saturation.

### Ethical consent

The Ethics Committee of the University of Venda provided approval for the study. Permission to conduct the study was also obtained from the Limpopo Provincial Department of Education and the principals of the participating schools. The participants were informed that participation was voluntary and that confidentiality of their information would be ensured. Permission was obtained from participants to voice record the focus group interviews. The participants were assured that their responses would not be linked to their personal identities and that after transcription the recordings would be destroyed five years after publication of the results. Consent was obtained from all participants in the study prior to the interviews. They were informed that if they needed counselling as a result of their participation in the study; arrangements would be made with a private counsellor. None of the participants sought counselling as a result of their participation in the focus group discussions.

### Trustworthiness

Trustworthiness measures the truth value of a study and is indexed by measures to enhance credibility, dependability and confirmability [24]. Credibility was achieved through prolonged engagement with the participants and establishing rapport. Dependability was ensured by the voice recorder and the transcripts which were available for the use of verification if it was necessary. Confirmability was ensured by conducting focus group discussions (FGDs) until data saturation was attained, and developing an audit trial which is a systemic collection of documentation. The documentation consisted of the field notes which the researcher took during the FGDs interviews, the voice recordings of the proceedings during the FGDs interviews and the verbatim transcripts that were done by listening to the voice recordings.

### Data analysis

Data were transcribed in Xitsonga and translated into English by a first language Xitsonga speaker. The accuracy of the translations was checked randomly by another first language Xitsonga speaking research assistant. The English and the Xitsonga versions of the interviews were then carefully read and checked by the first author as she had conducted the interviews and is fluent in both Xitsonga and English. The data were managed by means of the qualitative data management software, NVivo (version 8) using a general inductive approach. The predefined themes were explored and an inductive process was used to derive subthemes from the main themes. As coding occurred, a ‘tree structure’ was generated in which themes and subthemes were linked to one another. The findings are structured according to these themes and subthemes. RATS guideline for reporting qualitative studies were adhered at all points during the study.

## Results

### Knowledge on warning signs of suicidal behaviour

Participants were asked if they had observed any sign of poor mental health in the deceased students. All the teachers in the five groups mentioned that they had not observed any change in the students’ behaviour preceding their deaths. *“You may find that the signs were there, they were just beyond my comprehension, just like in the case of another teacher who stated yesterday that she noticed her and she asked her why she was always sleeping when she gets into the class, but she did not know why she was sleeping, it was only after she died that she thought it means that the reason why she was sleeping was because she had a problem,”* said a male teacher.

Although there were some signs which some teachers observed such as sleeping during lessons, they had not identified them as warning signs of the suicide. This unusual behaviour of the students was not associated with problems that the students could have had.

### Perceptions on cause of the suicide

Teachers were asked if they knew what factors had caused the learners to commit suicide. Teachers in all the focus groups stated that they did not know the reason for the suicide as the students had not confided in them or in their peers. They mentioned other factors that they thought could have contributed to the suicide of the learners, such as broken relationships, pregnancy, the use of drugs, young people not wanting to be reprimanded, a mother having a relationship with another man, child-headed households, the culture that prevented young people from discussing sexual issues with parents, evil spirits and black magic, family history of suicide, and HIV/AIDS. The teachers did not mention themselves as potential factors in the students’ suicides. Teachers indicated that as adult guardians and teachers, they were not able to respond to learners’ emotions, especially when they seemed to have love and relationships difficulties. They explained that they were unable to provide the needed guidance and counselling.

“*If students approach us as their parents or teachers and confide in us, we usually say that their romantic issues have nothing to do with us, we say that they are still at school or are too young to be concerned with love, and we dismiss them*”, said a male teacher. *“Students are not free to come and discuss those problems with us, or we are not seen as parent figures in the school where learners feel free to contact us for guidance and counselling,*” said a male teacher.

### Discussing the suicide of a student in class

The majority of the teachers pointed out that they did not talk about the death of a peer to students. *“Yes, there is nothing from my side, I do not know from other members of management, there was no single day where I took action, where I made a conscious effort to try to talk to children about it,”* said a deputy principal.

Teachers seemed to be afraid to face students after a suicide, because it seems as if they did not know what to say or how to discuss the death of the student. Only two teachers in the five groups indicated that they went back to their students to discuss with them about this issue, even though it was not on the day that the suicide took place. The first teacher said, *“I was trying to advise those children and asked them to imagine how they would feel if they found themselves in a situation similar to that of the deceased. I told them that they should not see such situations as being the end of their lives. I suggested that they should try to deal with circumstances by talking to a very close person, or a teacher at school. I told them that we were not just their teachers, but their parents as well. What I can say is that I tried after the incident, not on the very same day because I was so scared, but on another day”*. The second teacher mentioned: *“The classes that I entered were not coping. I just comforted them by saying that it happened and it cannot be reversed. What we need to do is to accept it”.*

Some teachers in the five groups stated that the principals of the schools took the initiative and called an urgent meeting with teachers and students. One of the principals, a clergyman, held a meeting with both teachers and students soon after receiving the death message. His position as a clergyman enabled him to counsel teachers and students and to bring the situation at the school under control.

### Blaming teachers for the suicide

Some teachers mentioned the utterances made by community members at a memorial service of a deceased student that had affected them emotionally and had made them angry. One teacher said: *“When I attended her memorial service I heard some people referring to the suicide, but they touched on the incident while directing criticism at her Life orientation teacher.* (Life orientation is one of the subjects done by learners at school. The focus is on the personal, social, intellectual, emotional and physical growth and development of learners). *They suggested that children were not taught about these things. We then indicated that teaching has to do with teaching someone so that he will pass to the next level. Some people are able to take what you teach and implement it in the practical situation,”* said a male teacher.

### School curriculum content related to suicidal behaviour

In responding to the question, “Which content on suicidal behaviour do you teach the students?” teachers mentioned that there was no information on suicidal behaviour in the school curriculum. Information included in the subject Life orientation dealt with stress, HIV/AIDS, and sexual harassment, but did not include issues related to suicide. Teachers reported that they were not trained to deal with suicidal students. Therefore, it is not surprising to see teachers displaying little knowledge of the warning signs of suicidal behaviour.

### Resources available to help survivors of suicide

Regarding available resources, teachers said that there were no services available at schools to assist suicide survivors (teachers, students, and administration staff) following a student’s suicide. Some participants mentioned that although there were no services at schools, they were fortunate because their principal was a clergyman. The skills he had used in making the announcement of the student’s death combined with the way he delivered the scripture from the Bible, had reduced the high emotions that had prevailed among the teachers and students. The following quote has been taken from the transcripts, “*The principal comforted the children by mentioning the fact that everyone should understand that in life we will come across undesirable situations in one way or the other. Your father may pass away or your mother may pass away, you may be the one who passes away or your brother or any person who is very close to you. So if it is something that we always know and accept that such things can happen, when it happens it will not hurt us so severely because we know that such things do happen in life. So, I felt comforted that while we need to be aware of this issue, something undesirable will sometimes happen when you are less expecting it, not everything that will happen according to your plan”.*

### Prevention of suicide among high school students

The teachers were asked for their opinions on how to prevent students committing suicide. A male teacher said: “*Yes, I understand all that my colleagues said to me when that incident occurred. It was the second at this school since I started teaching there, the first suicide had been committed by a male. I then asked myself how do we deal with these kinds of children or all the children? What is it that we do from time to time to ensure that we can help them and prevent these things? I wondered how it would be if some people could be tasked to convene gatherings, male teachers with male students and female teachers with female students, to try to talk to them about leading a positive life”*. Another teacher said: *“We should also consider the issue of religion. Religion is able to transform a person’s behaviour and be able to stand strong even when he or she has to face difficult situations. It may only be difficult because these days, there is freedom of religion. Because in terms of religion, a young person is not allowed to engage in sexual activity before the appropriate time arrives”*.

The teachers offered several suggestions with regard to suicide prevention of high school students. Some thought that it would help if students could be educated and could create realistic self-perceptions because they would learn to accept themselves and what they had. They thought this could be addressed by including the issue of suicide in the school curriculum. Teachers further suggested that counsellors should be stationed at schools. The fact that religion plays an important role in shaping an individual’s behaviour was also emphasised. They argued that if students did not engage in sexual activity before marriage they would not fall pregnant which in turn could reduce the stress. As religion makes people to be strong, raising children in a religious way can assist students to cope with difficult situations appropriately.

## Discussion

The present study assessed the knowledge, views and training needs of high school teachers regarding students’ suicide in the rural areas in South Africa. Focus group discussions were used to enable the researcher the opportunity to observe if the teachers were comfortable discussing the topic among themselves. The discussions with teachers in Limpopo showed that information on suicidal behaviour was not included in the school curriculum. It became apparent that teachers were not well informed about the precursors of suicidal behaviour and how best to respond to them. Participants in the focus groups expressed the need to be able to identify students who indicate a tendency towards suicide and refer them to relevant professionals for help. More specifically, findings showed that high school teachers displayed a lack of knowledge of the warning signs of suicidal behaviour. Students presented warning signs before they died, but teachers were not aware that these were warning signs or did not know what the behaviour meant. Research has shown that adolescents who have attempted or completed suicide show warning signs in advance. As mentioned above, these warning signs include talking about committing suicide, developing eating or sleeping problems, withdrawing from friends or social activities, giving away prized possessions, having made previous suicide attempts, taking unnecessary risks, losing interest in personal appearance, and using of alcohol or drugs [12,13]. Therefore, because teachers spend many hours with the students they need to enhance their knowledge of these warning signs, and their ability to respond effectively to them. In this way teachers might be able to help to decrease suicide rates among high school students in South Africa [17,25,26].

Teachers were unaware of the cause of the students’ deaths. They were able to guess what the possible causes could have been. None of the teachers in our study blamed themselves for the students’ death, even though some appeared to have been unfriendly towards students and sent them away with their problems unresolved. Some teachers were aware that students were not free to consult them when faced with problems because they did not trust them as it seems to be common for teachers to discuss students and divulge information entrusted to them; such behaviour could strain their relationships with students. This notion is supported by Shilubane et al. [10] and Thanoi [27] who found that relationship problems were a determinant of suicide attempts and death by suicide among adolescents. As parents and teachers are the most important social support systems that adolescents have, failing to listen to them means that students have to keep their problems to themselves. A study by Bae and colleagues [28] demonstrated that social support promotes mental health and prevents mental problems, while Shilubane et al. [11] found that a lack of social support is an important contributor to suicide attempts among young people.

The association between drug use and suicidal behaviour has been established in several studies [14-16,29-33]. As drug use is perceived as one of the causes of suicide, measures should be taken in schools to prevent students from accessing drugs. In addition, teachers indicated a need for programmes that focus on the development of adolescents’ skills in dealing with socio-emotional problems. The present school curricula focus on academic achievement and not on the development of such skills. A study by Chen and Li [34] established that prevention and intervention programmes focusing on adolescents’ emotional functioning, such as depressed moods, might help them improve their social as well as their academic achievements.

The majority of the teachers in the study did not discuss the death of a student by suicide with fellow students in class. Teachers seemed to be afraid to do this, probably because they do not know how to manage the emotions of their students. Only two teachers talked about suicide, but they did not go to the class and initiate a discussion. It can be assumed that some students needed counselling but did not get the service they needed [35]. Such students could have been identified by teachers during class discussions about the suicide and if needed, the teachers could have referred them to a psychologist for counselling. It could be possible that the teachers’ behaviour of not wanting to confront the students might be associated with not knowing how to handle students when emotions erupt. Indeed, this confirms the findings of some other studies [12,17] that professionals actually received little training in the handling of difficult clinical problems.

The discussions with teachers in this study showed that information on suicidal behaviour was not included in the school curriculum. Teachers were not well informed of the precursors of suicidal behaviour or how best to respond to them, and expressed the need to be able to identify suicidal students and refer them to relevant professionals for help. If the training of teachers and the content included in Life orientation were to be reviewed to include information on mental health and suicide, teachers would be aware of the warning signs of suicidal behaviour and they would be more likely to take appropriate action. The perspectives of teachers would be very important for the future development of interventions because they are able to play an important role in prevention of suicide.

In addition, services and resources are needed at schools to deal with students at risk and prevent suicides as well as to handle the emotional well-being of survivors. Participating teachers reported that community members were angry and sometimes blamed them for the students’ suicides. This made teachers angry, as they felt that community members had questioned their teaching. Some teachers indicated that community members had voiced concern as they did not expect to see a death of student by suicide while Life orientation was taught at school. Studies suggest that the anger displayed by community members and the blame attached to teachers is a normal reaction when a death has occurred, especially if the death has not been of natural causes [36]. According to WHO [7], South Africa is a middle-income country with few resources; it is not surprising to find that there are no services in Limpopo province schools to deal with suicide related behaviours. Lower-income and middle-income countries have lower budgets for health services in general and for mental health in particular. More budgets are allocated to address the Millennium Development Goals (MDGs) while mental health receives less attention. As a result, there are few sustained efforts activities that focus on suicide prevention [7]. Thus it can be concluded that theory and evidence-based school-based interventions involving training of teachers in identifying symptoms of psychosocial problems that may lead to suicide and skills in handling such problems is required [12].

## Conclusion

Despite the limited generalizability of the present study because of the small sample and the specific geographic location under study, it is the first study that focused on the role of teachers in suicide in Limpopo province. The research reported in this article has provided an insight into the needs of high school teachers with regard to prevention and counselling for those involved. Furthermore, it contributed to our understanding of suicide in South African adolescents and provides guidelines for the practice of adolescent suicide reduction through school-based education programmes. In order to inform the development of school-based suicide prevention programmes more quantitative research is needed to establish representative findings on knowledge, skills, and training needs of teachers and on available services in and for schools with respect to student suicide. Such information may contribute to the development of theory and evidence-based school-based interventions [37] that aim to prevent and identify health problems among adolescents and contribute to the reduction of suicide among adolescents in South Africa. This research should be extended to other areas of South Africa to make implementation at the national level feasible and in line with specific contexts.

Also, the focus group discussion proved to be an appropriate data collection method for this study because it gave the researcher the opportunity to observe the teachers if they were comfortable discussing the topic among themselves, and eventually with their students. Should they have displayed uncomfortable behaviour, it would have assisted the researcher in making assumptions that a suicide prevention program focusing on teachers might not be effectively implemented should it happen to be developed, as teachers would not be comfortable discussing the topic with students as displayed by their unease feeling among themselves.
